# Limited Effects of Low-to-Moderate Aerobic Exercise on the Gut Microbiota of Mice Subjected to a High-Fat Diet

**DOI:** 10.3390/nu11010149

**Published:** 2019-01-11

**Authors:** Filipe M. Ribeiro, Camila F. A. Ribeiro, Ana C. M. Garcia, Alinne P. Castro, Jeeser A. Almeida, Octavio L. Franco, Bernardo A. Petriz

**Affiliations:** 1 Post-Graduation Program in Physical Education, Catholic University of Brasilia, Brasilia 71966-700, DF, Brazil; filipemouraudf@gmail.com; 2 Center for Proteomic and Biochemical Analysis, Post-Graduation in Genomic and Biotechnology Sciences, Catholic University of Brasilia, Brasília 71966-700, DF, Brazil; ocfranco@gmail.com; 3 University Center—UDF, Research Group of Molecular Exercise Physiology, Brasilia 70390-045, DF, Brazil; anacaiana@gmail.com; 4 S-Inova Biotech, Catholic University Dom Bosco, Biotechnology Program, Campo Grande, 79000-000, MS, Brazil; camila.faribeiro@gmail.com (C.F.A.R.); palinne@gmail.com (A.P.C.); 5 Programa de Pós-Graduação em Saúde e Desenvolvimento na Região Centro Oeste—PPGSD, Faculdade de Medicina—FAMED, Universidade Federal de Mato Grosso do Sul, Campo Grande 71966-700, MS, Brazil; jeeser@gmail.com

**Keywords:** gut microbiota, obesity, low-to-moderate intensity, physical activity, diet

## Abstract

Several studies have indicated that diet and exercise may modulate the gut microbiota in obese subjects. Both interventions were shown to alter the microbiota orthogonally. However, this relationship has not been fully explored. This study analyzed the effects of low-to-moderate aerobic training on the fecal microbiota of mice subjected to a high-fat diet (HFD). Here, 40 male mice (C57Bl/6) were divided into two groups with standard diet (SD; 12.4% lipid) and HFD (60.3% lipid) for four months. These groups were divided into four, named SD control, HF control, SD trained and HF trained. All animals were submitted to an incremental test to estimate low-to-moderate maximum speed. Training consisted of 30 min·day^−1^, 5 days/week, for 8 weeks. The HFD increased the body weight (*p* < 0.0001) and adiposity index (*p* < 0.05). HFD also negatively influenced performance in exercise training. Moreover, the diversity of gut microbiota was reduced by the HFD in all groups. A low-to-moderate exercise was ineffective in modulating the gut microbiota composition in mice subjected to HFD. These findings suggest that two months of low-to-moderate exercise does not achieve a preponderant modulatory effect on shaping microbiota when submitted to the high-fat diet.

## 1. Introduction

Obesity is one of the most impactful chronic nontransmissible diseases in the world [1]. To date, about 13% of adults are obese, and 39% are considered to be overweight worldwide [2]. Also, the obesity pandemic has resulted in high public health expenditure [3] and a high death rate worldwide [4]. Due to the high pathogenesis complexity, significant efforts are being made by public health policies and scientific research to establish novel strategies in its prevention and treatment [5]. 

Studies have shown that the gut microbiota plays a significant role in the pathogenesis of obesity [6], where the positive regulation within its content may be used as a clinical treatment [7,8,9]. The gut microbiota consists of millions of microorganisms responsible for a series of physiologic functions related to the maintenance of host homeostases, such as immunologic maturation within the gut, digestion of complex polysaccharides, and protection against pathogens [6,10]. Diverse factors such as host–genotype interaction, environmental factors [11], pathogens [12], antibiotics [13] and lifestyle [14] are also known to shape the gut microbiome. However, Western diets, marked by high caloric values and high fat content, are one of the well-known factors in the induction of obesity [15]. Lately, a series of studies have shown that these obesogenic diets also modulate the gut microbiota, leading to a specific microbiota profile, with less diversity, thus with greater facility in harvesting energy from digestion and accumulating fat [15]. Interestingly, the transplantation of fecal microbiota from lean subjects to obese individuals seems to be effective in enhancing its diversity and composition [16]. In this context, the modulation of the gut microbial consortia is being investigated as a possible novel strategy in the treatment of obesity.

Furthermore, and in parallel with dietary manipulation, physical activity was also shown to induce alterations within the composition, abundance and gut microbiota diversity in experimental models [17] and humans [18,19]. To this end, a series of exercise stimuli have been used to assess the role of exercise in the modification of the gut consortia, which include high- [20] and moderate-intensity training [21,22], and a comparison between voluntary and forced exercise [23]. This last study indicated that both stimuli distinctively alter the gut bacteria, which must be considered for further interpretations. Nevertheless, only a few studies have investigated the role of exercise and dietary intervention in obese subjects [8,24]. Taking these facts into consideration, the shaping of the gut microbiota through exercise may be one alternative strategy for the prevention and treatment of obesity [10,25]. However, there are no studies about the effects of low-to-moderate exercise on gut microbiota, even less when associated with the previous induction of a high-fat diet (HFD).

Both the gut microbiota and exercise have been implicated in obesity [10,26]. However, the effect of controlled low-to-moderate exercise training on gut microbiota of mice that were induced by an HFD remains unexplored. For this reason, in the present study, the composition of gut microbiota and the training intervention were evaluated using the 16S rDNA gene sequence from fecal samples. Due to the growing research into the effects of exercise training on gut microbiota of obese subjects [17,27], we hypothesized that a protocol of eight months of exercise training performed at a low-to-moderate intensity (50% of maximal velocity) would play a significant role in gut microbiota modulation by HFD induction. Our results, described below, show the limited effects of exercise on the obesity context by altering gut microbiota.

## 2. Material and Methods

### 2.1. Animals

Animals were obtained from the Bioassays Laboratory of the Catholic University of Brasilia (UCB), and all animals (40 isogenic male mice, C57BL6) started the experiment at ~4 weeks of age. The mice were individually allocated in separate cages to avoid the “cage effect” (animals kept in the same environment tend to have similarities in their microbiota) [28]. The bioterium temperature was maintained at 23 °C (±2 °C) and kept in a 12 h light–dark cycle environment. The Animal Use Ethics Committee (CEUA) of the Catholic University of Brasilia, Brazil, approved the methods and conduct that were used in the experiment (UCBDOC, no. 026/15). After experimental procedures, animals were anesthetized with 2% xylazine (50 mg·kg^−1^) and 10% ketamine (80 mg·kg^−1^) and euthanized by cervical dislocation. All procedures were performed following the relevant guidelines and regulations as here described. Through the entire experiment, all efforts were made to minimize animal suffering, and a veterinarian, and a technician specialized in Confined Animal Facilities, accompanied all the procedures described below.

### 2.2. Experimental Design and Exercise Training

Right after weaning, the 40 animals were divided into two groups: a group fed with a standard diet (SD, 68.8% kcal as carbohydrate, 18.8% kcal as protein and 12.4% kcal as lipids) (*n* = 20) and a group fed with a high-fat diet (PRAGSOLUÇÕES Biociências, BRA), (HFD, 21.3% kcal as carbohydrate, 18.4% kcal as protein and 60.3% kcal as fat, *n* = 20) as described by Cano, Santacruz [7]. The objective of this process is to induce chronic obesity in approximately 4 months of feeding on the HFD [29]. All animals received water *ad libitum* throughout the experiment. The animals’ body weight, as well as the weekly intake (in grams), were weighed by an analytical balance (Shimadzu, AUY220) throughout the experiment. Also, the caloric value of the weekly intake was calculated by the value in grams ingested per week (g/week). Furthermore, after euthanasia, the adipose tissue was collected, and total body fat was measured by the calculation of the adiposity index (AI %; the sum of the weight of retroperitoneal, subcutaneous epididymal and omental white adipose tissue/body weight × 100) [30]. 

**Figure 1:**
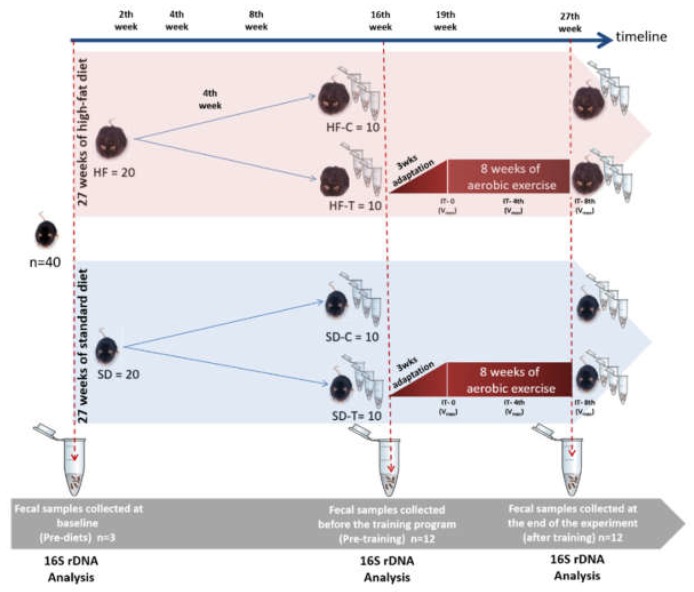
Experimental design. C57BL6 male mice (*n* = 40) were divided into two groups: high fat (HF) and standard diet (SD). Afterward, animals were subdivided into four groups: standard diet control (SD-C, *n* = 10); standard diet trained (SD-T, *n* = 10); high-fat control (HF-C, *n* = 1); and high-fat trained (HF-T, *n* = 10). IT-0 (incremental test before training), IT-4 (IT at the 4th week of training), IT-8 (IT at the 8th week of training). Fecal samples were collected in the following periods: pre-diet, before training, and after 8 weeks of training.

After 4 months of diet-induced obesity, and before exercise training, all animals were submitted to an initial acclimatization and adaptation process in the environment where the training took place. The treadmill (Treadmill Exer 3/6, Columbus Instruments, Columbus, OH, USA) used can force exercise by a mild electronic shock grid (0.2 mA) at the rear of the treadmill. The acclimatization processes occurred on the treadmill with no movement from 10 to 30 min for five days. After that, the animals were adapted for three weeks with time and velocity being increased up to the limit of 20 m·min^−1^, as described by Almeida et al. [31,32]. After the adaptation period, the mice were randomly distributed into four groups: standard diet control (SD-C, *n* = 10); standard diet trained (SD-T, *n* = 10); high-fat control (HF-C, *n* = 1); and high-fat trained (HF-T, *n* = 10) (Figure 1). An incremental test of maximum velocity (IT-V_max_) was performed in three periods: before training, after 4 weeks of training and after the end of the training protocol (total of eight weeks of training). The V_max_ was performed on a treadmill running with the start of the test at 6 m·min^−1^ and with increments of 3 m·min^−1^ every three minutes until exhaustion of the animal. The objective of this test consists of the evaluation of the V_max_ and how the evaluation was individualized. All animals performed the IT-V_max_. After an evaluation of the V_max_, the animals trained for 2 months at an intensity relative to 50% of the V_max_, five times a week with duration of 30 min per session (Figure 1). Control groups remained untrained for 8 weeks; however, these groups ran the IT-V_max_.

### 2.3. Fecal DNA Extraction and 16S rDNA Gene Sequencing

Three fecal samples of each animal were collected during the following periods: pre-diet (absolute control), before training and after 8 weeks of training. The fecal collection was performed 24 h after the last bout of the exercise session. Samples were immediately stored in RNAlater^®^ solution (Life Technologies, Carlsbad, CA, USA) until stored at −80 °C. Fecal microbial DNA was extracted from ~0.25 g using the PowerFecal DNA Isolation Kit (MoBio, Carlsbad, CA, USA) according to the manufacturer’s instructions. The triplicate DNA extractions were not pooled together, being analyzed individually. Hybridization of the conserved region of the 16S rDNA gene occurred with the use of the regions V3 (5′–CCTACGGGNGGCWGCAG–3′) to V4 (5′–TACHVGGTATCTAATCC–3′) to the bacterial domain, generating a fragment with ~444 bp. However, for these fragments to be compatible with the MiSeq Illumina sequencing platform, specific adapters were coupled: 5′–TCGTCGGCAGCGTCAGATGTGTATAAGAGACAG-3′ (V3F) and 5′–GTCTCGTGGGCTCGGAGATGTGTATAAGAGACAGGAC-3′ (V4R). Fragments of the 16S rDNA genes were amplified via polymerase chain reaction (PCR), in this order: microbial DNA (5 ng·µL^−1^), amplicon PCR Primer 1 μM, amplicon PCR Reverse Primer 1 μM and 2× KAPA HiFi HotStart ReadyMix with a final reaction volume of 25 μL. The reaction cycle was carried out with an initial denaturation of 3 min at 94 °C, followed by 35 cycles with denaturation for 30 s at 94 °C, annealing for 60 s at 50 °C and last, extension for 90 s at 72 °C, followed by a final extension from 7 min to 72 °C and cooling to 10 °C. Subsequently, the PCR products were quantified via Qubit^®^ (Life Technologies) and then sent directly to BPI Genotyping—Botucatu/SP, for the preparation of the amplicons, such as the attachment of the barcodes with the Nextera^®^ XT Index Kit and running the run on the MiSeq Illumina platform, according to the standard protocol (16S Metagenomic Sequencing Library Preparation).

### 2.4. Analysis of 16S rDNA Sequences

All of the 16S rDNA amplicons were processed by the quantitative insights into microbial ecology (QIIME) pipeline version 1.9.0-dev. [33]. The sequences obtained were filtered for quality control and grouped into operational taxonomic units (OTUs) using 97% similarity. Briefly, the phylotypes were grouped at the species level using UCLUST as standard parameter [34]. Samples were used for the rarefaction analysis to determine alpha diversity. The principal coordinate analysis (PCoA) of unweighted UniFrac distances was calculated and compared between the period of pre-diet (absolute control), before training (SD-C and HF-C) and after training (SD-T and HF-T) to observe the similarities in the composition of the gut microbiota. Beta diversity and PCoA analysis were also calculated within the QIIME using unweighted UniFrac distances. 

### 2.5. Statistical Analysis 

Statistical differences of the samples were based on a measure of distance in the PCoA plot using the analysis of similarities (ANOSIM) by permutation of group membership with 999 replicates [35]. The statistical test of the taxonomic differences between the samples was calculated by the ANOVA variance analysis with the Tukey test to calculate the confidence interval (nominal coverage of 99%) and comparison of the means, using the Statistical Analysis of Metagenomic Profiles (STAMP) software version 2.0.0 [36]. Statistical analysis of alpha diversity and other physiological variables (e.g., body weight, food intake, adiposity index, and running performance) were performed on GraphPad Prism 5.00 (ANOVA with Tukey–Kramer post-hoc test considering *p* < 0.05). The experiment was conducted just once.

### 2.6. Accession Number

The Illumina MiSeq read data for all samples, which have been deposited in the National Center for Biotechnology Information (NCBI) Sequence Read Archive database, project number: SRP130142.

## 3. Results

### 3.1. Effect of High-Fat Diet and Low-to-Moderate Exercise on Body Weight, Adiposity, Food Intake and Calorie Consumption 

**Figure 2:**
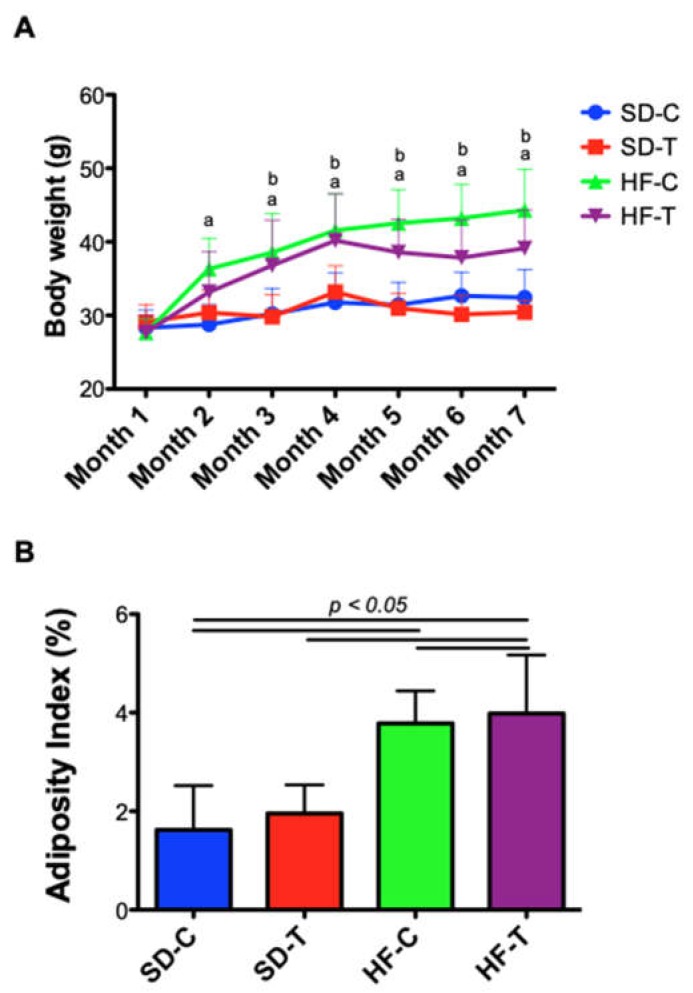
Effects of diets and exercise on body weight and adiposity index. (**A**) Two-way ANOVA was used to verify differences of body weight over eight weeks. The “a” means difference between control groups (SD-C vs. HF-C) at the same time. The “b” indicates differences between the trained groups (SD-T vs. HF-T). There was interaction between “diet x exercise”, *p* < 0.001, F = 3.31. (**B**) Significant differences between control groups compared to exercise groups (*p* < 0.05, represented as a line).

**Figure 3:**
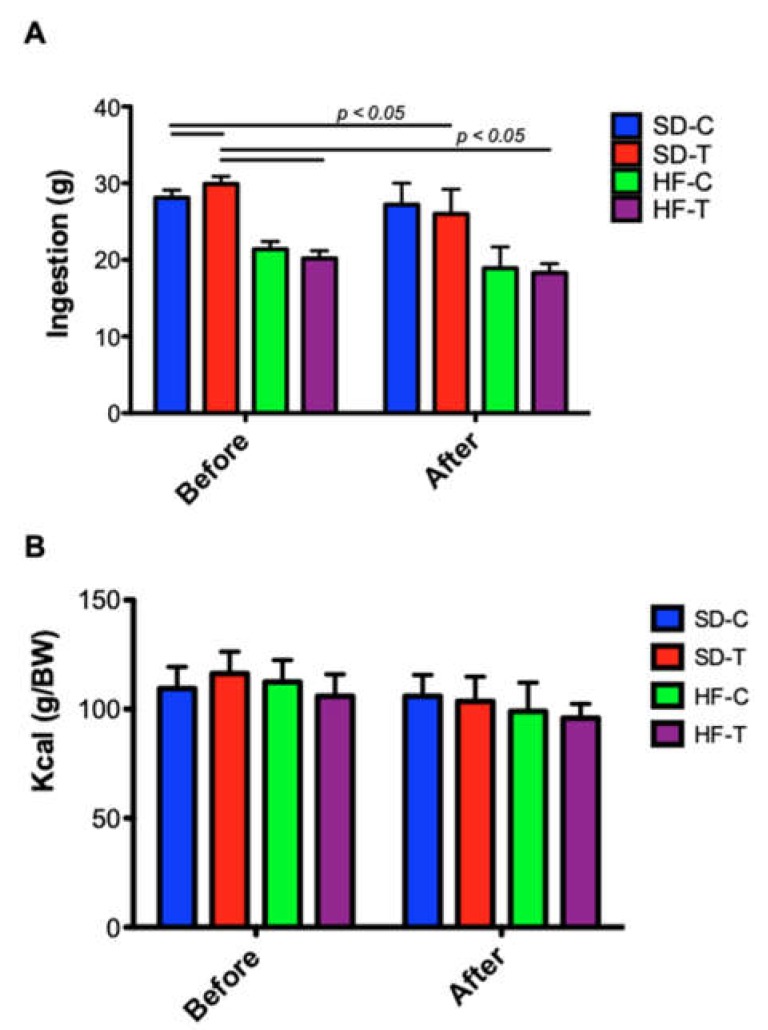
Food intake and kilocalories ingested by all animal groups. (**A**) Significant differences of food intake between the control groups when compared to respectively exercised groups (diet vs. training, *p* > 0.05, diet, *p* < 0.05, and training, *p* < 0.05). (**B**) There is no difference in caloric intake between groups.

During the diet administration (when compared to the first and last week: pre-diet vs. 27 weeks), a significant weight gain was observed in the animals fed with HFD when compared to those fed with SD (mean difference of 36.7% of the weight, *p* < 0.0001). The control groups SD-C and HF-C presented a significant difference from the second month of ongoing diet (*p* < 0.0001), being represented by the letter “*a*” (Figure 2A). The trained groups (SD-T and HF-T) had significant differences from the third month (*p* < 0.01), being represented by the letter “*b*” (Figure 2A). High-fat groups had higher levels of adipose tissue compared to standard diet groups (Figure 2B). Interestingly, there were no significant changes due to physical training at a low-to-moderate intensity (50% of IT-V_max_). There were significant differences in the food intake between the control groups SD-C and SD-T vs. HF-C and HF-T before training (*p* < 0.0001) and between the trained groups SD-C and SD-T vs. HF-C and HF-T after training (*p* < 0.01) (Figure 3A). Also, no difference was observed in caloric intake (kcal) from all experimental groups (Figure 3B). Thus, the caloric intake in all groups was similar to their respective compositions (HFD with 60% lipids and SD with 10% lipids).

### 3.2. Effect of Low-to-Moderate Aerobic Exercise on Aerobic Power (V_max2_)

**Figure 4:**
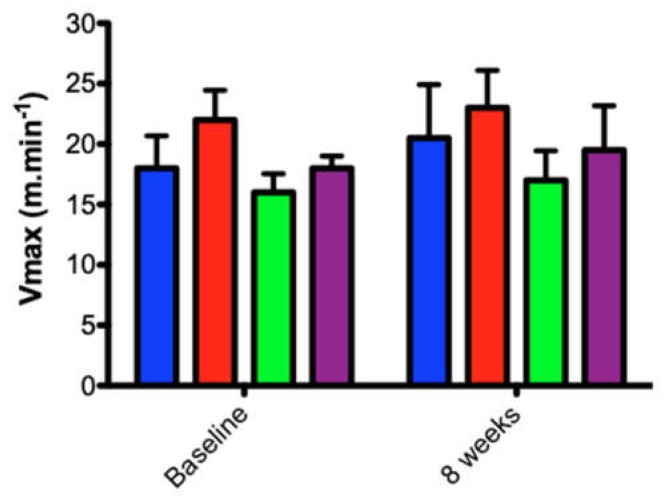
Incremental test. Two-way ANOVA revealed no significant differences between control and exercised groups (interaction, *p* > 0.05, diet, *p* < 0.05, and training, *p* < 0.05).

After eight weeks of treadmill running at a low-to-moderate intensity, corresponding to 50% of the IT-V_max_, two-way ANOVA revealed that no significant difference was observed in interaction (diet vs. exercise, *p* > 0.05) but with differences between moments (before vs. after), *p* = 0.04, and groups (*p* < 0.001, Figure 4).

### 3.3. Characterization of the Bacterial Community by Gut Microbiota Profiling

After quality filtering, due to the expected value discrepancy between regions during sequencing, it was necessary to normalize the samples, delimiting the number of minimum sequences for maximum utilization. Thus, analyses of α- and β-diversity were performed with samples that obtained a number greater than or equal to 113,854 sequences for each sample. Therefore, one of the biological replicates did not contain the minimum number of sequences mentioned above, which led to its exclusion from α- and β-diversity analysis.

**Figure 5:**
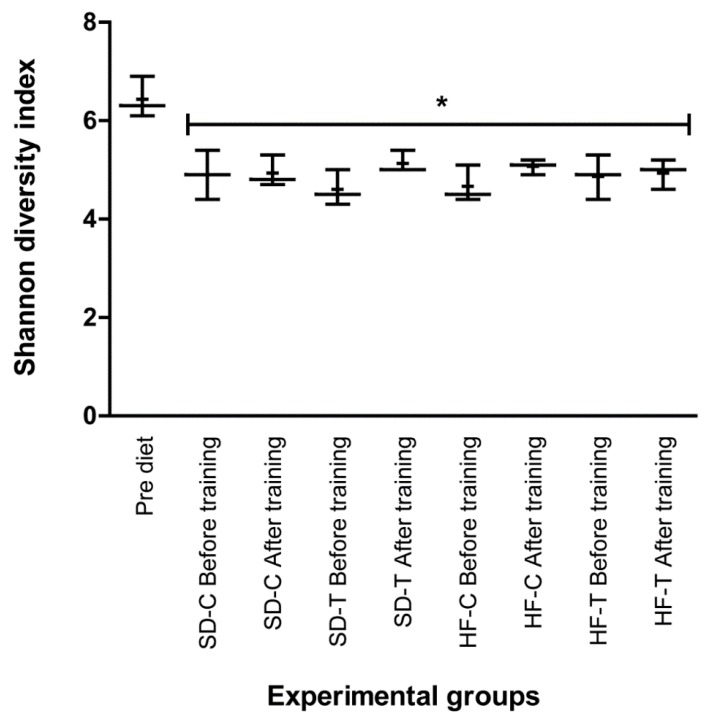
Bacterial alpha diversity. Comparison of alpha diversity estimation of the 16S rRNA gene libraries at 97% similarity from the sequencing analysis (ANOVA with Tukey–Kramer post-hoc test).

In this context, bacterial diversity was assessed by the rarefaction measure of observed species against the number of sequences per sample. The α-diversity analysis (Shannon-Wiener index) revealed that the OTU richness in pre-diet fecal samples is more species-rich than those found both in samples after four months of diet application, as well as after the training intervention (Figure 5). 

### 3.4. Principal Coordinates Analysis (PCoA)

**Figure 6:**
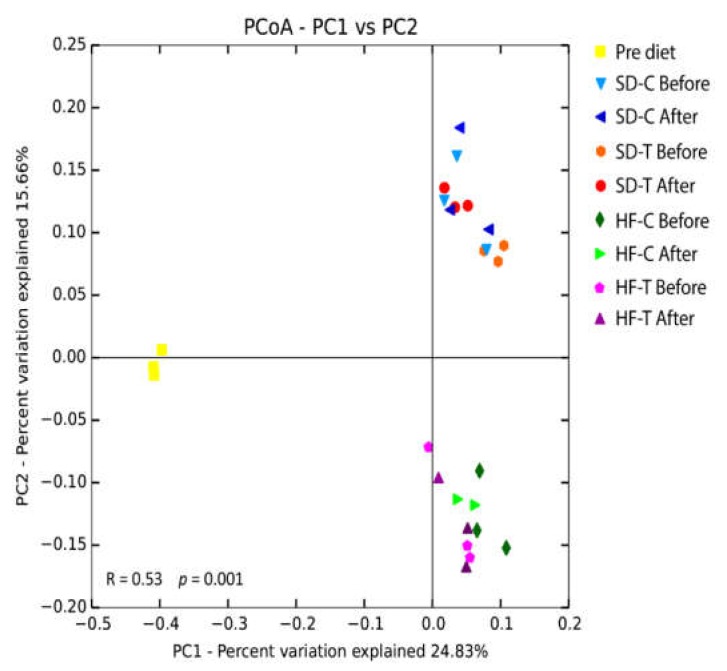
Effect of diets on the bacterial community. The PCoA of unweighted UniFrac distances was generated with the sequences obtained by the samples in the pre-diet (absolute control), and before and after training periods. The ANOSIM similarity analysis results show significant alteration of the gut microbiota by the diets.

Principal coordinates analysis (PCoA) of unweighted UniFrac distances aimed to compare the effect of diet and exercise on the bacterial community of all samples. Unweighted UniFrac (PCoA) analysis of the fecal samples presented a higher similarity of the bacterial composition due to the application of both diet interventions (HF and SD) (Figure 6). Here, animals that were fed with HD (before and after training) are plotted in the lower quadrant, and samples fed with SD (before and after training) are plotted in the upper quadrant (Figure 6). These data indicate that the diet application led to a different bacterial composition between the two diets (HFD and SD diets), both after four months as well as after low-to-moderate exercise (R = 0.53, *p* < 0.001). The time difference between periods (pre-diet vs. before and after training) also played a role in the distinct bacterial community compositions for these different groups. Accordingly, eight weeks of exercise training performed at a low-to-moderate intensity (50% of maximal velocity) did not cause reliable modifications in the composition of the bacterial community (R = −0.048, *p* = 0.79). 

### 3.5. Effects of Low-to-Moderate Exercise on Bacterial Diversity

**Figure 7:**
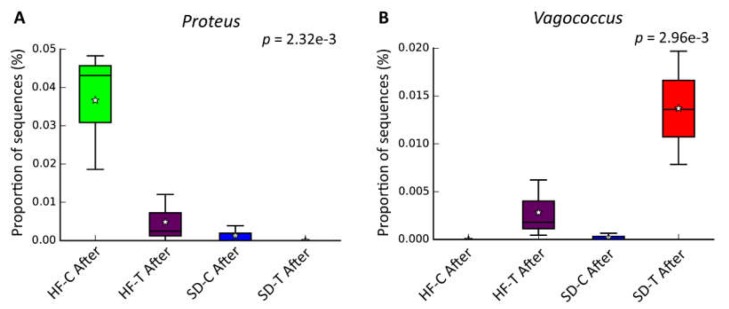
Effect of exercise training on bacterial genera profile. (**A**) *Proteus*; (**B**) *Vagococcus*. White star represents the average of each group, and the median value is shown as a line within the box. (*p* < 0.01 was considered as statistical significance, ANOVA with Tukey–Kramer post-hoc test.)

Concerning the modulation in the abundance of bacterial genera due to low-to-moderate exercise (95% confidence interval), *Proteus* and *Vagococcus* were shown to be altered (Figure 7). The comparison between all experimental groups indicated that those submitted to high-fat diet obtained greater expressions of the genera *Proteus* (HF-C and HF-T), especially the HF-C group (Figure 7A). These data indicate that exercise played a role in reducing *Proteus* expression. The standard dietary groups (SD-C and SD-T) presented lower expression of *Proteus*. Finally, the exercise protocol (SD-T and HF-T) was relevant in the expression of the genus *Vagococcus* (Figure 7B). Besides, when compared to the trained groups (SD-T and HF-T), it can be observed that the high-fat diet decreased the expression of *Vagococcus*.

## 4. Discussion

Throughout life, the gut microbiota is significantly modulated by several environmental and host-related factors [11]. In this context, dietary intervention is one of the most potent factors in shaping the gut bacterial community [8,15,35]. Studies have shown that a high-fat diet (HFD) negatively modulates the diversity and composition of bacteria in mice [35,36]. These changes within the gut microbiota were observed over eight, 12 and 16 weeks [36]. In accordance with the literature, in our study, 8 weeks of induction by HFD was accompanied by a significant increase in body weight and adiposity index (Figure 2), resulting in a lower bacterial diversity (Figure 6). 

Some studies discuss the effect of exercise, where exercise solely does not seem to be effective for weight reduction [37,38], especially in obese models (e.g., Zucker rats) [39]. Exercise has been partially beneficial in improving health in rodents consuming an HFD, whereas incorporating a “better” diet can ameliorate the metabolism and general health [38]. However, in our study, the protocol of eight weeks of low-to-moderate exercise was not able to reduce the weight gain and adiposity index among the HFD groups (HF-T vs. HF-C). Thus, these groups presented an improvement in weight and adiposity when compared to the student diets groups (Figure 2). Moreover, it was observed that HFD tends to alter food satiety, also de-affecting the process of appetite control [40,41]. According to our data, the ingestion (in grams) of animals fed with HFD was lower, when compared to the animals fed SD. However, although the caloric amount of HFD is higher than SD, the caloric intake of all groups was similar, with no statistically significant difference between them. Thus, the increase in weight gain (Figure 2) of the HFD groups may have been caused by different dietary macronutrient metabolism [42]. 

Following the incremental test of maximal velocity (IT-V_max_) (Figure 4), the HFD may have influenced the physical performance of the respective groups. This was verified by a better performance of the SD-C vs. HF-C (*p* < 0.05) and SD-T vs. HFD groups before training (*p* < 0.01). Also, there was no statistical difference between HF-T and HF-C after training. This adverse effect on running performance has also been seen in other studies [43,44]. The exposure to high-fat diets is associated with faster accumulation of blood lactate [43], reduction in lipolytic signaling pathways [45], disturbed glucose metabolism [46] and impairment of skeletal muscle mitochondrial function [44]. In our study, it is possible that these physiological aspects may have influenced the performance of the IT-V_max_ before the training period. In agreement with this, it was observed that the SD-C group also had a better performance before and after training, when compared to the HF-C group (*p* < 0.05) (Figure 4). Similarly, the SD-T group also continued to have an improvement in aerobic power compared to HFD groups after training (*p* < 0.05). However, beneficial results have been observed in athletes that consume an ideal dietary fatty acid composition, and it appears that induction by HFD is required for several months [47,48]. 

Regardless of diet, better cardiorespiratory fitness has been recently correlated with increased gut microbial diversity [49]. In this sense, animals fed HFD did not improve performance with training (Figure 4). However, other studies with different intensities such as high-intensity interval training (HIIT) [20] and endurance exercise showed that they have a different role in microbial diversity [50]. Our results showed an increase of the *Proteus* genus in high-fat diet groups (Figure 7A). It is noteworthy that the HF-T group obtained a lower expression of *Proteus* compared to the control (HF-C). This effect of exercise on the lower abundance of *Proteus* was also visualized in the standard diet group that trained (SD-T vs. SD-C). 

*Proteus* genus species have been commonly observed in the intestinal tract and considered as human opportunistic pathogens, where these bacteria are known to cause infection during impaired immunity [51]. Besides, elevated levels of *Proteus mirabilis* abundance have been associated with several inflammatory processes including inflammatory bowel disease and inflammatory arthritis and rheumatoid arthritis [52,53]. Furthermore, an increased *P. mirabilis* abundance was observed in rats that underwent a high-fat diet, also being significantly correlated with triglyceride levels, and leptin and insulin concentration, indicating that this bacterium may be associated with low-grade inflammation linked to obesity [54]. Some *Proteus* bacteria have been related to lipopolysaccharide (LPS) biosynthesis [55]. It is well known that elevated levels of LPSs are linked to inflammatory reactions in obesity [56]. Thus, as observed in our study, the lower abundance of the *Proteus* genus after prolonged aerobic exercise may be a positive indicator and control from low-grade inflammation associated with obesity. 

An increase in the abundance of *Vagococcus* genera was also observed in the trained groups (Figure 7B). This genus belongs to the *Enterococcaceae* family, well known for its bacteria that synthetize lactic acid as their primary metabolic end product [51,52]. Despite its unclear mechanism, lactic acid bacteria are known to have an anti-obesity effect [53,54]. Species such as *Lactobacillus gasseri* and Lactobacillus delbrueckii are reported for their effect on cholesterol metabolism [55]. Also, the administration of L. gasseri as a probiotic supplement was reported to reduce adiposity and body weight in obese adults [55]. The *Enterococcaceae* family was also associated with a low-fat diet (10% fat) in a study that investigated the effect of a high-fat diet and genetically induced obesity on the intestinal microbiome and metabolome of a mouse model for colorectal cancer [56]. Despite the increase of *Vagococcus* genera after training, more studies are necessary to further understand the anti-obesity effect of these bacteria. 

Although exercise has played a role in reducing *Proteus* and increasing *Vagococcus*, these data reveal that exercise with low-to-moderate intensity has a limited effect on gut microbiota. It is important to note that the time of only high-fat diet induction (16 weeks) was twice the time of exercise application (8 weeks). In this way, more studies with different protocols of physical training in contrast to the high-fat diet become necessary. Another factor that may have influenced the results is the intensity applied in the present training protocol. Our results show for the first time that low-to-moderate intensity was not able to counteract the effects of prolonged HFD induction. However, the experiments from our study were conducted just once, being a limiting point to be considered in this study.

## 5. Conclusions

The present study demonstrated that gut microbiota diversity was reduced by diet in all experimental groups. Despite being the first study to apply a low-to-moderate aerobic training protocol, this stimulus presented a reduced effect on gut microbiota shaping when contrasted with the high-fat diet used to induce obesity. However, other training variables such as intensity, volume and even other types of exercise stimuli must be explored, particularly when applied to pathologic models such as obesity. As recently reported by Mitchell et al. [57], rigorous dietary control in larger samples is essential to further identify the influence and mechanisms of exercise on the gut microbiota, and how these stimuli may be extended to novel clinical treatments of metabolic diseases such as obesity.
